# Understanding the Impact of Historical Policy Legacies on Nutrition Policy Space: Economic Policy Agendas and Current Food Policy Paradigms in Ghana

**DOI:** 10.34172/ijhpm.2020.203

**Published:** 2020-11-09

**Authors:** Anne Marie Thow, Charles Apprey, Janelle Winters, Darryl Stellmach, Robyn Alders, Linda Nana Esi Aduku, Georgina Mulcahy, Reginald Annan

**Affiliations:** ^1^Menzies Centre for Health Policy, School of Public Health, University of Sydney, Sydney, NSW, Australia.; ^2^Department of Biochemistry and Biotechnology, Kwame Nkrumah University of Science and Technology, Kumasi, Ghana.; ^3^Global Health Governance Group, Usher Institute of Population Health Sciences, University of Edinburgh, Edinburgh, UK.; ^4^Médecins Sans Frontières, London, UK.; ^5^Centre for Universal Health, Chatham House, London, UK.; ^6^Development Policy Centre, Australian National University, Canberra, ACT, Australia.; ^7^Kwame Nkrumah University of Science and Technology, Kumasi, Ghana.

**Keywords:** Policy Analysis, Food Policy, Public Health Nutrition, Political Economy

## Abstract

**Background:** The global food system is not delivering affordable, healthy, diverse diets, which are needed to address malnutrition in all its forms for sustainable development. This will require policy change across the economic sectors that govern food systems, including agriculture, trade, finance, commerce and industry – a goal that has been beset by political challenges. These sectors have been strongly influenced by entrenched policy agendas and paradigms supported by influential global actors such as the World Bank and International Monetary Fund (IMF).

**Methods:** This study draws on the concept of path dependency to examine how historical economic policy agendas and paradigms have influenced current food and nutrition policy and politics in Ghana. Qualitative data were collected through interviews with 29 relevant policy actors, and documentary data were collected from current policies, academic and grey literature, historical budget statements and World Bank Group Archives (1950-present).

**Results:** Despite increased political priority for nutrition in Ghana, its integration into food policy remains limited. Food policy agendas are strongly focused on production, employment and economic returns, and existing market-based incentives do not support a nutrition-sensitive food supply. This policy focus appears to be rooted in a liberal economic approach to food policy arising from structural adjustment in the 1980s and trade liberalization in the 1990s, combined with historical experience of ‘failure’ of food policy intervention and an entrenched narrowly economic conception of food security.

**Conclusion:** This study suggests that attention to policy paradigms, in addition to specific points of policy change, will be essential for improving the outcomes of food systems for nutrition. An historical perspective can provide food and health policy-makers with insights to foster the revisioning of food policy to address multiple national policy objectives, including nutrition.

## Background

The global food system is not delivering affordable, healthy diverse diets.^1,2^ Despite declines in hunger and undernutrition globally in the past two decades, 821 million people are undernourished, micronutrient deficiencies are widespread, and hunger is again on the rise.^3,4^ This trend is particularly concerning in the Africa region; since 2014, the number of individuals with moderate or severe food insecurity in the region has increased, with over half of the population now facing food insecurity. If recent trends continue the region’s distribution of undernourishment will be the highest in the world by 2030. Preliminary assessments indicate that the coronavirus disease 2019 (COVID-19) pandemic may further threaten attainment of Sustainable Development Goal 2.1 (Zero Hunger).^4,5^ At the same time, poor diets are contributing to an escalating prevalence of diet-related non-communicable diseases (NCDs), which are a major cause of death and disability globally.^6^ The economic cost of this double burden of malnutrition (sometimes referred to as a ‘triple burden’, to emphasise micronutrient deficiencies) have been estimated at $US3.5 trillion per year.^7,8^

Food system change to support access to nourishing food has been identified as a critical component of action on the double burden of malnutrition.^9^ However, in countries with a long history of hunger and undernutrition, addressing the emerging and co-existing epidemic of diet-related NCDs presents a significant policy challenge. This double burden of malnutrition requires a shift in food policy attention from “sufficiency” to encompass considerations of both adequate nutrition and nutritional quality relevant to NCD prevention.^10^ These considerations include the price and availability of healthier (minimally processed, nutrient-rich) foods, and the creation of food environments that support improved access to healthier foods relative to less healthy foods, particularly ultra-processed foods.^11^ This will entail new investments in production, storage, transport, trade and marketing, and thus require policy change across ‘food policy’ sectors like Agriculture, Trade and Industry.^12^

Uptake and implementation of global recommendations to improve nutrition through food system policy has been slow and patchy.^13,14^ In part, political challenges to public health nutrition policy change may be related to entrenched policy agendas and paradigms supported by influential global actors. For example, within the Agricultural sector, a persistent focus on cash crop production and export has undermined investment in traditional domestic agricultural production, contributing to a lack of capacity to meet demand for local staples and fresh foods in many low-and middle-income countries (LMICs).^15,16^ This policy focus is in part a colonial legacy, but has continued to be championed and extended by international economic agencies.^17,18^ Similarly, structural adjustment programs (SAPs) reduced the (historically high) taxes on agriculture, but also significantly reduced state investment in agriculture as part of broader efforts by the World Bank and other global institutions to reduce fiscal deficits and government expenditure.^19^ Experience in Malawi was that donor-supported SAPs concentrated on “promoting market and price mechanisms, less on addressing production constraints and non-economic barriers to broad-based economic growth.”^20^ These paradigms were entrenched in the following decades by institutional structures and political power that perpetuated the status quo.^21^ In India, the framing of nutrition as an economic concern by the World Bank had a profound influence on the uptake of ‘cost-effective’ nutrition interventions narrowly focussed on individual, rather than food system, changes.^22^ These historical influences on food policy point to strong economic agendas that have had a long-term influence on the way that food policy is conceptualized and understood, including limited integration of nutrition.

 Given the limited progress on nutrition-oriented food system transformation globally, we posit that an understanding of the historical context of current food policy – including legacies in both content and paradigms – can provide insights that would enable public health policy-makers at the global and national level to effectively support wide-scale food system policy change. This study examines how historical economic policy agendas and paradigms have influenced current food and nutrition policy and politics, using Ghana as a case study. Our aim is to shed light on historical structures and paradigms that limit the integration of nutrition considerations in food policy. In this study, we consider public policy as referring to specific statements of intent or action by the government, and politics as referring to actors, interests and power. We undertook this study from an explicitly public health perspective, seeking to identify opportunities to strengthen consideration of nutrition in food policy, such that access to affordable nourishing food is improved for the population of Ghana.

Ghana has made significant progress in reducing child undernutrition over the past 25 years, mainly due to poverty reduction. For instance, although stunting among children persists in Ghana, it has reduced significantly. Ghana is one of only five sub-Saharan African countries on course to reduce the number of stunted children by 40% by the year 2025.^23^ However, regional pockets of food insecurity remain, and the country now faces a double burden of malnutrition characterized by persistent high rates of micronutrient deficiencies, persistent stunting among children, and a growing prevalence of obesity and diet-related NCDs.^24^ In Ghana, SAPs in the 1980s had a significant impact on agricultural policies, and the World Bank played a key role in providing policy advice regarding both agriculture and nutrition.^25-29^ Ghana thus presents a relevant case study for improving understanding of how historical policy paradigms and agendas can shape current food and nutrition policy, and how food policy can better address the double burden of malnutrition.

## Key Messages

**Implications for policy makers**

- Integrating nutrition into food policy remains a significant challenge for policy-makers in Ghana and globally.
- An entrenched liberal economic paradigm and narrowly economic conception of food security, associated with structural adjustment and trade liberalization remain influential in the food policy sectors.
- Understanding the historical policy context of food policy can provide insights for engagement by health policy actors across policy sectors, to enhance integration of nutrition considerations into food system policy.

**Implications for public**

Food is largely the policy responsibility of economic sectors of government, including Trade, Agriculture and Commerce. However, there has been limited consideration of nutrition in these sectors – even in a context like Ghana, in which nutrition is a clear policy priority for the Government as a whole. In this study, we have analysed the historical context of food policy in Ghana, in order to better understand the drivers of policies that affect food systems. The findings of this study provide insights for strategic advocacy for policy to support healthier and more sustainable food systems. In particular, they can help identify policy opportunities and ways to explain the importance of integrating nutrition considerations that take into account the mandates and paradigms of economic policy-makers.

## Methods

This paper presents a historical policy space analysis of food policy in Ghana, with a focus on the post-independence period, drawing on qualitative case study research methods.^30^ Data were collected through interviews with relevant policy actors, and from policy-relevant documents.

The aim of our analysis is to identify where current food policy in Ghana, which spans multiple sectors, reflects historical policy legacies and to use this information to inform learning for future policy change to address the double burden of malnutrition. As per Hall’s theory of social learning, we thus considered (1) the role of ideas and frames as a potential conduit for policy legacies and (2) policy change at the ‘order’ of policy paradigms and policy instruments.^31^ Policy paradigms refer to the “framework of ideas and standards,” including linguistic, normative, and epistemic dimensions that underpin policy goals and selection of instruments (ie, specific ‘types’ of policies, such as taxation, or public education).^31^ We also drew on policy space analysis to guide design of our study and research instruments, in order to examine dynamics within the cross sectoral food policy subsystem. ‘Policy space’ refers to the “freedom, scope, and mechanisms that governments have to choose, design, and implement public policies to fulfill their aims.”^32^ Policy space analysis focuses on the interplay between context, policy characteristics and agenda-setting circumstances in understanding dynamic policy evolution in a policy subsystem.^33,34^

###  Framework for Analysis

This study is grounded in the concept of ‘path dependency’ from an historical institutionalist approach to policy science, which in a policy context refers to the way in which choices made can narrow conceptually the set of subsequent choices available to decision-makers.^35,36^ It does not suggest that the past in any way predicts the future, but acknowledges that present policy is the sum of both accumulated policies over time and ‘new’ decisions. Both of these may carry echoes of previous decisions, due to factors such as inherited institutional structures (both normative and positive), sunk costs (real or perceived), the privileging (or otherwise) of certain interest groups over others, and the establishment of commitments or contracts (in this case, perhaps related to structural adjustment or development aid).^35^ We chose to ground the study in path dependency because its institutional orientation complemented the policy analysis frameworks focused on policy learning, and is consistent with Hall’s explicit acknowledgement of the influence of policy legacies.^31^ It also supported our objective of interrogating influences on policy content over time in their paradigmatic and institutional context. In addition, the evidence available to inform current policy may also be the product of long-term investments in certain types of intervention or historical preferences for certain types of data (eg, quantitative or outcome data compared to process oriented or qualitative data), as seen in global health metrics more broadly.^37^

###  Data Collection

 We conducted 23 interviews with policy actors in Accra and Kumasi, Ghana, between February and April 2018. Twenty-nine policy actors were interviewed; four interviews had two interviewees, and one had three. Interviewees had experience in Health (n = 6), Agriculture (n = 12) and Economic/Industry (n = 13) sectors (two interviewees had both nutrition and agriculture experience). Eleven interviewees were women and 18 were men. Five people who were invited to be interviewed declined, four from Economic/Industry sectors, and one from Agriculture. Interviewees included: past and present food policy-makers and implementers in the Government of Ghana (n = 19); personnel of non-government organizations (NGOs) representing public and private interests (n = 3); academics (n = 3) and staff of international agencies relevant to food policy in Ghana (n = 4). Interviewees were identified through purposive sampling of food policy-relevant agencies and snowball sampling. Interviewees were recruited through formal letters of invitation to relevant agencies and individuals (where retired or independent).

 The interviews were semi-structured and designed to elicit information on potential historical legacies that are evident in the current policy environment. Specifically, the interview schedule asked about (1) population experiences of food and the food system, (2) changes to the food system, and (3) food policy in Ghana, in relation to the current situation, and changes over time (the past 50 years). Interviews lasted 45-80 minutes and were recorded and transcribed in full, except for two interviews for which permission to record was declined. Two of the interviewers took detailed notes for these interviews, which were written up in detail immediately afterwards.

 We collected current Government of Ghana health, agriculture and food sector policy documents through searches of government websites, and direct requests to relevant government ministries. We identified government budget statements as a key source of information regarding significant historical policy decisions and paradigms (since 1960). We obtained these for the various years available from government websites and the government archives in Accra. We also identified relevant information by searching the archives of the World Bank for historical documentation on Ghana related to food and agriculture (based on title), both online and in hard copy. In addition, we searched for existing literature on historical food policy in Ghana in Google Scholar, using the following search terms: food, policy, Ghana, agriculture, World Bank. Documents identified are cited in the Results. We searched the online archives of the International Monetary Fund (IMF) and Food and Agriculture Organization (FAO) but identified little information of relevance.

###  Data Analysis – Coding and Initial Analysis 

We analysed the interview data thematically, using qualitative data management software (NVivo^TM^) to organize the data related to both current and historical policy. Our study frameworks formed the basis for pre-determined codes, augmented with open coding (Table 1).

**Table 1:** Coding Framework

**Predetermined Codes (Deductive)**	**Open Coding (Inductive)**
Actor interests; actor power; policy commitments (domestic and international); evidence; food system change; frames; historical policy paradigms and approaches; ideas; mechanisms of influence; institutional relationships; institutions (current and historical); nutrition; policy instruments, paradigms and settings; previous interventions; sunk costs	Stakeholder engagement; education and training; jurisdictional responsibilities; policy priorities; agriculture; development paradigm

**Figure 1:**
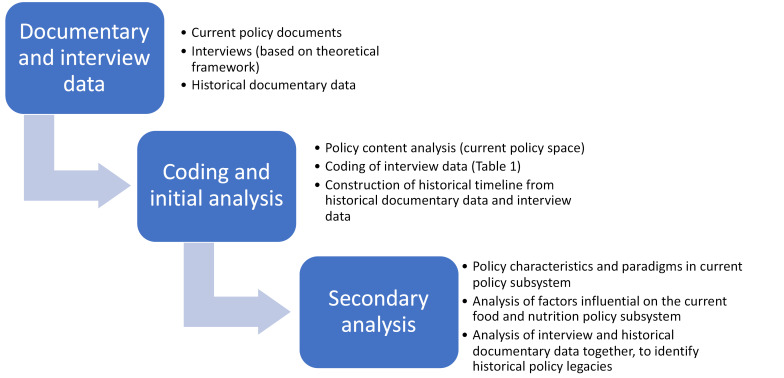
Summary of the Data Collection and Analysis Process.

We extracted relevant data from current policy documents relevant to food and nutrition into a content analysis matrix in Microsoft Excel^TM^, based on our study frameworks. The data extracted included: policy objectives, content (instruments and settings/policy characteristics) relevant to food and nutrition, and evident framing of food and nutrition as policy issues (Figure 1).

We extracted relevant data from historical policy documents and budget statements into a content analysis matrix in Microsoft Excel^TM^, based on our study frameworks, to document dominant policy frames/paradigms, instruments and settings over time.

 Finally, we created a table, organized by decade (1960s to present), to document relevant information on government policy paradigms and priorities from the literature, economic policy statements and World Bank documents.

###  Data Analysis – Secondary

 We analysed the data iteratively, with the aim of successively focusing our data collection as indications of potential historical legacies were identified, based on our research objectives.

 We first analysed current policy across sectors with respect to policy characteristics within this subsystem, as well as evident paradigms and objectives related to nutrition and food systems, drawing on the policy content analysis and interview data relevant to current policy characteristics. This analysis provided us with a baseline from which to investigate historical ‘policy legacies’ evident in current food system policy with respect to nutrition. The first two sections of the Results present the findings regarding nutrition and food policy characteristics.

Second, we analysed the coded interview data to understand the underlying agenda setting circumstances (namely frames and beliefs about the policy space and its origins), context and institutional structures influencing *current* policy; in particular, in relation to the evident separation of nutrition from food system policy. This analysis indicated that major factors reflected in current food system policy are beliefs about nutrition, a long-term historical policy focus on food as an economic commodity, and the institutional and policy context. The third section in the Results is structured in line with these findings (Figure 1).

 Third, we analysed the coded data and the historical documentary data together, to identify historical policy legacies. This analysis focused on: (1) beliefs and frames regarding the nature of the ‘food system problem’ and how these have changed (or not) over time; and (2) current priorities, power and institutional structures in the policy space, and their historical antecedents. These legacies are explained in the fourth section of the Results, with documentary sources cited.

## Results

###  Overview of Findings

 In the results, we first examine the integration of nutrition and food as policy issues in Ghana. Analysis of the current policy frameworks and interviews with key stakeholders indicated that nutrition was a clear policy priority for the Government of Ghana. However, responsibility for achieving nutrition objectives was located primarily within the Ministry of Health and there was limited integration of nutrition as a consideration in the governance of food more broadly. We then present our findings regarding the historical evolution of food policy over time in Ghana, for insights as to the roots of this limited integration. We identified three historical policy legacies that help to explain the persistent lack of integration of nutrition into food policy in Ghana.

###  Current Nutrition Policy in Ghana

At the time of this study, nutrition was a clear policy priority for the Government of Ghana. The review of current policy documents and interviews indicated widespread recognition of the importance of nutrition for development, and high-level political commitment to improving nutrition within the *Medium-Term National Development Policy Framework - An Agenda For Jobs: Creating Prosperity And Equal Opportunity For All (First Step) 2018-2021.*^38^ This National Development Policy Framework clearly identified the double burden of malnutrition as a policy priority for Ghana, and includes food and nutrition security as a social development goal.^38^ It also explicitly identified the need to address nutrition through both nutrition-specific (generally health sector led) responses and food system oriented (nutrition-sensitive) responses. However, the emphasis in the National Development Policy Framework is on undernutrition. This emphasis is likely also compounded by Ghana’s membership of the Scaling Up Nutrition Movement (since 2011), which is led by the National Development Planning Commission and focusses on undernutrition and nutrition-specific interventions. This focus on undernutrition as the main nutrition issue in Ghana was echoed in the interviews; particularly in the Agriculture and Economic sectors, undernutrition was identified as the main nutrition challenge.

At the sectoral level, we found explicit nutrition commitments in the health and agriculture sector (Table 2). The Health sector had developed a comprehensive *National Nutrition Policy*, which addresses undernutrition, micronutrient deficiencies and overweight and obesity, as well as food security and food safety.^39^ The policy activities are broad and multifaceted but (understandably) focused on nutrition-specific interventions delivered by the health sector, which does not have a mandate to address the food system.

**Table 2:** Summary of Current Policy Priorities Relevant to the Food System

**Sectors**	**Current Policy Priorities Relevant to Food and Nutrition**
National development	Nutrition is a priority, and nutrition-specific (health sector) and nutrition-sensitive (agricultural production and reductions in post-harvest losses) are both identified in the ‘social development’ section.^38^ Food and agriculture are addressed in the ‘economic development’ section. Expanding food production, agro-processing and exports are a priority, with a key objective being improving rural livelihoods and creating employment.^38^
Health	National Nutrition Policy addresses all forms of malnutrition through nutrition-specific and nutrition-sensitive interventions; Agriculture and food security is included as a priority, including to facilitate access to diverse foods, and enhance nutrition across the food system.^39^
Agriculture	Promote employment in agriculture, through links to markets, productivity gains, and post-harvest management.^40^ Nutrition addressed in a “sub-programme of nutrition sensitive agriculture” which includes: food fortification; education on dietary diversity and consumption of biofortified crops; enhancing production and consumption of livestock breeds.^40^
Trade	Promotion of: Agro-processing and agricultural exports^41^ Export led industrialization strategy^41^ Domestic market-led industrialization strategy based on import competition^41^ The explicit food focus is restricted to standards (ie, promoting quality as per food safety)^42^
Industry	A priority is agro-based industrial development; linked to efforts to reduce poverty. Food as one ‘input’ commodity for industry – efforts to improve consistency of supply.^43^
Fisheries	Ensure appropriate management and use of fish stocks; Improve fisheries management; Reduce post-harvest losses. Improving food security is a goal of the Management Plan, but not reflected within the objectives and no further reference.^44^
Local economic development	Promote local economic development and competitiveness; Strengthen trade associations and co-operatives; Improve infrastructure.^45^

Nutrition was addressed in Agriculture sector policy through a sub-programme of nutrition-sensitive agriculture spelled out in the government flagship programme, *Planting for Food and Jobs. *This programme included food fortification; education on dietary diversity and consumption of biofortified crops; and enhancing production of livestock and dairy.^40^

###  Current Food Policy in Ghana

**Figure 2:**
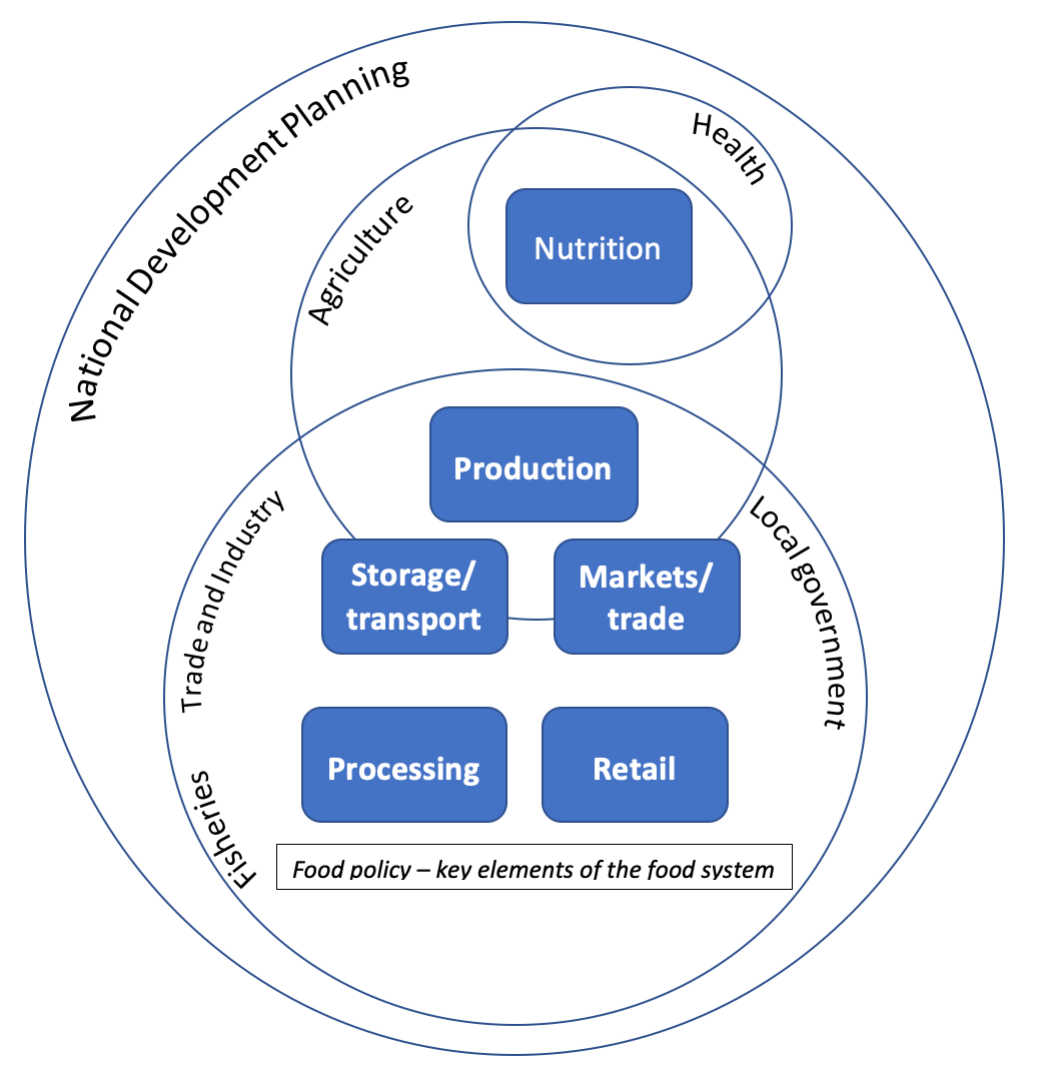
Summary of NUTRITION and Food Policy Responsibilities (Based on a Simplified Food System Structure) Across Ministries in Ghana. Source: Authors’ analysis; see detail on policies analysed in Table 2.

Policy directly related to food in Ghana is primarily located in the Ministry of Agriculture (production), Ministry of Trade and Industry (agro-processing, retail, trade and marketing), Ministry of Fisheries (production, processing and trade) and Ministry of Local Government and Rural Development (agriculture development and local industries) (Table 2). The National Development Planning Commission provides central government oversight across policy sectors, with guidance on overall national policy priorities (Figure 2).

Analysis of policy documents indicated that Agriculture policy objectives are oriented towards economic and livelihood development (Table 2). Six of the eight key objectives in the Agriculture policy focus on agriculture development, livelihoods, production quantity (including reducing post-harvest losses), and one objective was to promote animal production for income generation as well as food and nutrition security. The Ministry of Agriculture’s flagship programme *Planting for Food and Jobs* was mentioned by about half of the interviewees. The programme objectives include a focus on the main food commodities, with the objective of food security, as well as commercialization of agriculture, value chain development, export diversification and job creation. Interviewees reiterated that sector priorities were focused on livelihoods, productivity expansion and price stabilization. Four interviewees from Agriculture and Economic sectors noted that commercialization of agriculture and productivity expansion has been the main Agriculture sector priority for the past decade, with reference to financial investments in the sector. For example, “large scale commercial agriculture is priority, can see in funding for the Commercial agriculture projects, this is continuing in recent years” [NGO, Agriculture, #13]. This economic focus of agriculture is also reflected in Ministry of Fisheries policy, and also Ministry of Local Government of Rural Development policy and programming, which interviewees reported as supporting agricultural production through decentralized governance structures, as well as development of agribusiness as part of Small and Medium Enterprise development, but not addressing nutrition (Table 2). In addition, price stabilization has been a major priority due to the impact that prices have on consumer access to food. It appears that links between food security, food price and food inflation have contributed to raising the priority of price stabilization measures. For example, recent investment in the buffer stock programme was explicitly linked to food security: “[the] buffer stocks objective [is] to use buffer stock mechanisms to guarantee prices. … It used to be price stabilization but now it is moving towards food security as well” [Government, Economic, #6].

 There was little consideration of the nutritional value of food production, aside from the sub-programme which laid emphasis on nutritional value including through specific investment in nutritious orange-fleshed sweet potato and animal production. This marginalization of nutrition within the priorities of the Agriculture sector were explained by interviewees as arising from an understanding that food security, rather than nutrition, was core to the role of the Agriculture sector. For example, “[Agriculture] policy focuses much more on food security. We didn’t emphasize on nutrition.” [Government, Agriculture, #9]. This focus on food security, generally at a national level, appeared to reflect an understanding of the ‘problem’ as one of hunger and undernutrition, rather than access to nourishing foods. A Health sector respondent reflected on the focus on food supply, rather than nutrition priorities: “[Priorities are] mainly a supply-based thing. We don’t want to have shortages of rice or shortages of any food item; corn among other things which are even imported sometimes. I don’t think people are focused on healthy foods” [Government, Health, #7].

Policies of the Ministry of Trade and Industry also govern food, but do not consider nutrition. Current priorities relevant to food are to promote agro-industrial development (industry) and agro-processing (trade), and to increase production volumes and quality – particularly, consistency of production for local industries and export (trade and industry) (Table 2). These priorities were reflected by interviewee observations on the importance of food-related industrialization for achieving national economic and employment goals. For example: “Industrial transformation agenda is a huge priority, including improving the regulatory environment to promote business in food, agriculture and manufacturing … this is part of government’s agenda to create more jobs in the country” [Government, Economics, #3]. Food was also identified as a major opportunity to increase diversity in exports, a core strategy for achieving economic objectives. For example: “Horticultural products like banana, pineapple, cashew constitute most of the non-traditional exports, so agriculture is a priority for GDP [gross domestic product] growth” [Government, Economic, #1].

There was also a perception that large commercial actors were disproportionately influential in food policy. Six interviewees contrasted the relatively significant influence held by the (commercial) private sector with the minimal influence of smaller producers and industries. Another noted that smallholder farmers were overlooked in policy, in preference to commercial farmers: “When they started the *Planting for Food and Jobs* program, we did a strong campaign against what they intended to do in the beginning. The initial plan was to target what they call resource-rich farmers and those are farmers with 5 acres (2 hectares) of land and above so that means that they were leaving about 89% of Ghanaian farmers who own less than 5 acres of land” [NGO, Agriculture, #13].

 A characteristic of food policy discourse was the use of economic metrics to describe the ‘policy problem’ related to the current food system. Food system challenges were identified as being economic in nature (with very limited mention of nutrition) by 11 interviewees (seven in agriculture, three in economic sectors and one in health). These included a lack of local markets (particularly a local food processing industry), linked to a lack of value addition and processing of perishable produce, limited and unreliable transport and storage facilities, and limited access to finance and insurance for farmers leading to increasing risk and reducing profitability. Agro-chemical misuse was raised as a concern by seven interviewees.

###  Lack of Integration of Nutrition Into Food Policy 

In this section, we analyse the nature and framing of the lack of integration of food and nutrition policy, as the basis for our subsequent analysis of historical policy legacies. The National Development Planning and Health sectors in Ghana have supported nutrition as a policy priority – and are championing a multisectoral approach. However, integration of nutrition into food policy remains patchy. At the whole-of-government level, despite food being identified as important for both nutrition and economic growth, there was no explicit connection made between the nutrition section and the agricultural and economic sections of the Development Policy Framework. This was reflected in descriptions of food policy by the interviewees as primarily focused on economic objectives. Five interviewees from across sectors noted specifically that nutrition was a priority, but only for the Health sector, and not within sectors related to food (Table 1). Only a few interviewees linked nutrition challenges explicitly to the food system. One health sector interviewee identified food scarcity as associated with undernutrition, and one agriculture sector interviewee identified seasonality and lack of value addition as hampering access to nutritious foods.

 There appear to be two key factors underlying this separation of food from nutrition, which are fundamental to understanding the lack of integration of nutrition into food policy. First, nutrition was understood by interviewees as a health issue, and food as an economic issue. For example, “When you consider the way our system works, issues on nutrition are treated under health which helps organizations that deals with health and nutrition directly. Agriculture on the other hand focuses on crop and animal production and this does not promote togetherness with respect to work” [International Institution, Economic, #15]. This technical paradigm for nutrition has potentially contributed to the separation of food and nutrition. In addition, nutrition has evolved as a separate issue from food security, with nutrition a health issue and food security a national sufficiency issue. Food security is tied to economic objectives – further marginalizing nutrition in Agriculture sector priorities. For example: “food security [drives food policy in Ghana]. Even though with this new government’s flagship “planting for food and jobs,” the food is still leading. We want to generate more income to buy more food” [Academic, Agriculture, #5]. Second, the economic importance of the food sector in a national context in which economic concerns are positioned as the overriding priority of government, has meant that this dimension of food policy has become the primary mandate of Ministries of Agriculture, Fisheries, Trade and Industry, and Local Government and Rural Development. This sectoral imbalance towards economic concerns was given as a reason that developing an integrated policy encompassing food and nutrition was challenging; “Initially, the idea was to have food and nutrition security policy in 2011 but there was an issue that nutrition would be hidden under the food aspect because it wasn’t given much attention, so there was a need to separate nutrition from food” [Government, Economic, #6]. Such policy separation is mirrored by institutional structures that address food separate from nutrition. Interviewees indicated that there were economic sector committees that included agriculture, but that the Health sector were not included and thus nutrition did not form part of the considerations. Donor committees also perpetuate this, with interviewees reporting committees on “agriculture” as separate from those on “nutrition.”

 Compounding this separation of food and nutrition, it was also evident that governance responsibilities for “food,” from a food systems perspective, were not clear. Functionally, these responsibilities were split across ministries; a major point of separation was between food production (Agriculture and Fisheries) and trade and value adding activities such as processing (Trade and Industry; Local Government and Rural Development), while there was little specific governance of marketing and retail. These silos have resulted in the lack of an agency with a mandate to consider the multiple policy objectives served by food systems, such that an integrated policy framework (ie, including nutrition) could be developed. This was highlighted specifically by at least two interviewees as an approach that had been tried, with limited success. For example: “The situation is such that, the way the sectors operate, so we don’t really have one broad policy on food so to speak, but then there are specific components that guide production, processing etc. In the past, we’ve tried to get a more comprehensive but also coordinated food policy and that has really not taken off” [Government, Health, #12]. Economic policy-makers perceived that value addition was a neglected area in food policy, and that the long-term policy focus had been on production without similar attention to post-harvest storage, transport, and processing –contributing to poor economic outcomes. For example: “…the idea has been “Let’s grow,” “let’s produce” and once we produce, there would be market. For any commercial activity, the most important thing is the market” [NGO, Private Sector, #21]. There also appeared to be a tension between strategic government intervention to achieve food system objectives (eg, livelihoods, industry growth) and global agendas supporting liberal economic approaches, including trade liberalization. For example, an interviewee from the private sector commented: “When you want to grow some industries, you have to get some control to allow the industry to grow. These are some of the policies we want to implement but the World Trade Organization would say otherwise” [NGO, Private Sector, #21].

 Finally, the policy documents and interviews indicated that there was a separation between policy design and implementation that exacerbated the lack of integration between nutrition and food policy. Decentralization has resulted in much of the district-level implementation of policy being the responsibility of the Ministry of Local Government and Rural Development, including related to agriculture and local industry. The primary mandate for the Ministry of Local Government and Rural Development is economic, and it does not have a mandate for nutrition. In addition, three interviewees noted the mirroring of siloes at the point of implementation as a result of alignment between development agencies and siloed ministries. For example, “…there are key economic ministries like [trade and industry], food and agriculture, fisheries who are not working together like the way they should … Moreover, this incoherence is because developmental partners who support us pick specific ministries to work with. For instance, when Ministry of Food and Agriculture has funding to roll out a program, it is taken over by these partners and implementation is carried out from their perspective” [Government, Economic, #3]. Around a third of interviewees identified development partners (donors) as an influence on food policy in Ghana. In particular, donor funding was seen as being very selective, and that government often chose to align with donor priorities in order to obtain funding. As one agriculture sector interviewee noted: “Yes … [there is a strong donor influence] on our whole agricultural development. The strategy is not calling for that, but unfortunately, when you have your framework you can go in for funds to support technical capacity building and others” [Academic, Agriculture, #5]. Two interviewees mentioned the influence of donor commitments to global agendas, such as supporting large agricultural multinationals.

###  Historical Legacies of Food Policy in Ghana

 In this section we explore the persistent lack of integration of nutrition considerations into food policy, despite strong political commitment to addressing nutrition, from an historical perspective that draws on path dependency. In doing so, we identified three key historical policy legacies, from the 1960s onwards, that shed light on the persistent challenge of integrating nutrition considerations into food policy in Ghana.

####  Legacy 1: An Interventionist Approach Does not Work

 The interviews indicated that the perceived historical failures of an interventionist approach to food policy were still present in institutional memory. This historical legacy may implicitly act as a barrier to the adoption of a more coordinated or regulated approach to food policy because of the association with interventionist approaches to agriculture.

**Figure 3:**
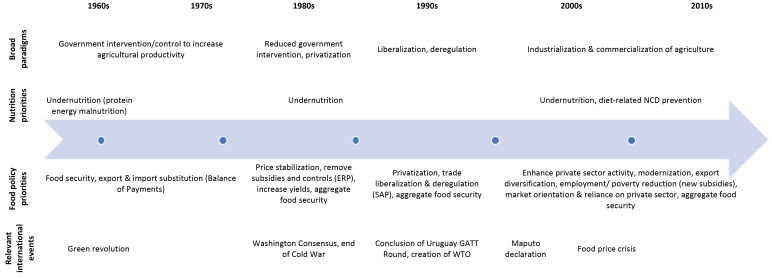
Overview of Food Policy Paradigms and Nutrition Priorities in Ghana Over Time. Abbreviations: NCD, non-communicable disease; ERP, Economic Recovery Program; WTO, World Trade Organization; SAP, structural adjustment program; GATT, General Agreement on Tariffs and Trade. Source: Authors’ analysis.

From independence and through the 1960s and 1970s, Kwame Nkrumah’s government and subsequent military governments adopted a highly interventionist and controlled approach to agriculture, including price controls, subsidies, and heavy state involvement in production, distribution and marketing (Figure 3).^46-48^ Key objectives during this period were increasing agricultural production, import substitution, and price stabilization.^46,48-50^ Efforts towards import substitution responded to World Bank/IMF recommendations to address the (im)Balance of Payments.^51^ As one interviewee described: “In terms of policy in the 60’s, 70’s, we had a plan. For instance, in the 70’s the plan largely talked about was ‘Operation Feed Yourself’ … we had [limited] subsidies on virtually everything” (International Institution, Economic #15). Operation Feed Yourself emphasized self-sufficiency and increasing productivity, following the Green Revolution in Asia, as well as export-oriented agriculture and supplying raw materials for local industry.^48,52^ It also had import substitution as a core objective, with a strong focus on production of staples, including maize, rice, yams, fruits and vegetables, oils, meat and fish, with minimum prices introduced for some crops.^53^ By the mid-1970s the focus had shifted towards industrial and cash crops such as rubber, sugar-cane, cotton, oil palm and groundnut, as well as commercial livestock and fisheries; but there remained a strong protectionist policy approach, with widespread import restrictions in place.^54,55^

This interventionist paradigm was widely recognized to have failed to achieve food security and economic goals. This was likely due to a combination of the complex bureaucratic nature of the intervention (including regional zoning and multiple layers of government involvement), as well as a strong overarching cash crop and export focus of agricultural support and limited consideration of marketing and distribution strategies for food.^56,57^ By 1980 Ghana was struggling to meet basic food needs and accepted a 12.7 million dollar loan from the United States of America for production of agricultural commodities, including wheat flour, corn, sorghum and rice.^58^

####  Legacy 2: Food Policy Should Adopt a Liberal Economic Paradigm

The interviews and analysis of historical documents revealed a major shift in the agricultural paradigm following the failure of the interventionist approach. This was characterized by a more liberal economic approach to food policy, that included privatization and reductions in policy support for the agriculture sector, and an increased focus on the role of food policy in achieving economic objectives related to livelihoods and economic growth (Figure 3).

In the early 1980s, following drought and bushfires from 1981-1983 as well as rising economic concerns, Ghana commenced a World Bank Economic Recovery Program (ERP).^46,59^ The ERP was implemented in three phases: stabilization, rehabilitation of the economy, and economic liberalization and development.^46,49,60^ Price stability and food security were main goals for the Agriculture sector during the first two phases, with the assumption that reduced inflation and increased commodity prices paid to farmers would lead to poverty alleviation.^61^ The third phase saw increasing emphasis on agricultural exports, with deregulation of commodity and service markets, liberalization of export markets, and reduced agricultural intervention, including removal of minimum prices and subsidies.^46,49,62^ It also included liberalization of imports and financial markets’ to attract investment.^62,63^ This final phase was associated with a shift in the agricultural paradigm to a more neoliberal approach characterized by scaling back state intervention and increasing private sector activity, because the State was seen as the problem, not the solution.^48,49^

This paradigm shift was noted by six interviewees with economic and agricultural expertise, who recalled the 1980s as the period that a shift to a ‘liberal economy’ began, with an emphasis on export-oriented agriculture that included limited investment in food and a focus on (macro)economic outcomes. During this time, there was a focus on training and capacity building of agricultural workers and policy staff by international agencies, including the World Bank, to support this new agricultural paradigm.^47^ Three of the interviewees also raised concerns about the reduced investment in agriculture during this period of the ERP and SAP that followed. For example: “I don’t think [structural adjustment] helped agriculture that much because there was a shift in investing in agriculture … there wasn’t much done” [Government, Economic, #2].

Agriculture sector reforms continued in the early 1990s with SAP III, focused on private sector development, privatization of State Owned Enterprises, removal of all input subsidies, phasing out of state intervention in the supply of inputs and farmer services, continued removal of trade restrictions, and improvements in trade facilitation.^46,64,65^ Agricultural policy objectives continued to focus on increased production – particularly for export – and food security^59,66-68^ This focus on production and export was reflected in comments by interviewees. Three interviewees noted agricultural export promotion and diversification in the late 1990s to include non-traditional exports (such as horticultural products). They all noted that key objectives during this period were poverty reduction and economic growth, reflected in the 1994 Agricultural Sector Development Policy; as one agriculture sector interviewee stated: “one of the issues was the need to increase income and economic growth and of course agriculture has always been seen as a key driver of the economy … there has been a lot of policies being put in place to increase agricultural productivity, but this is mainly for economic growth and income creation” [International Institution, Agriculture, #16]. The priority given to the private sector in the policy reforms during the 1980s and 1990s, as the engine of economic growth, were perceived as shifting the focus to the private sector. One interviewee described a situation in which large importers and wholesalers had successfully lobbied the government to reduce investment in production in the late 1990s (in order to increase imports).

The entrenchment of a liberal economic paradigm continued into the 2000s (Figure 3). With support from the World Bank, Ghana followed the SAPs with two Growth and Poverty Reduction Strategies during the 2000s. These were aimed at encouraging transformation from an agriculture-based economy to an industrial economy.^66^ Core Agriculture sector objectives were: strengthening the agro-industrial economy and stimulating private enterprise; export diversification; modernization of agriculture; food security; import substitution; increased productivity; and job creation.^49,69^ These priorities were reflected in the objectives of the Food and Agriculture Sector Development Policy (2003), which continued a market and private-sector orientation.^46^ Despite commitment to the Maputo Declaration in 2003 (to increasing national expenditures on agriculture and rural development to 10% of all budgetary expenditures) and a more coordinated approach to the Agriculture sector, Government expenditure on agriculture remained extremely limited.^46^

####  Legacy 3: Nutrition Is a Health Sector Policy Issue, While National Food Security Is an Agriculture Sector Policy Issue

 The third policy legacy relates to perceptions of food-related policy mandates. It was evident from historical documentation and the interviews that the divide between food and nutrition was also characterized by a compartmentalization of the role of the Agriculture sector as one of ensuring national food sufficiency and income generation through a production paradigm, and nutrition being a Health sector responsibility.

This has two facets. One is the long history of undernutrition as a nutrition priority in Ghana (Figure 3). By the 1970s, undernutrition had become a technical issue with a donor focus on technical health interventions, such as supplemental feeding and micronutrient supplementation – which entrenched the lack of attention to the food system within the health sector. Two interviewees (from Agriculture and Health) observed nutrition ‘solutions’ driven by donors had consistently focus on imported nutritional supplements rather than food system approaches that included agriculture and local crops.

The second facet was the narrowing of the food policy mandate to food security (at the aggregate national level), and away from a nutritional perspective from around 1970. In the early interventionist years, Kwame Nkrumah’s government implemented interventions to diversify diets and increase availability of nutritious foods to address the high levels of undernutrition at Independence (particularly protein-energy malnutrition, ‘kwashiorkor’). This included heavily controlled and interventionist agricultural approaches, such as setting up regional factories for (canned) meat and vegetables. However – in line with push back against the heavily interventionist approach, by the late 1960s food policy had shifted to focus on food security, defined as national food sufficiency and narrowing to staple crops. As one interviewee described: “Subsequently, when the military government regimes started, they focused on food security but not the composition of the food basket” [Academic, Economic #19]. This emphasis on national sufficiency and food security focused on staple crops as an objective for food policy was further fostered by World Bank initiatives in the 1970s and 1980s.^54^

## Discussion

 The dominant approach to food policy in Ghana at the time of this study was focused on economic policy objectives, and there have been persistent challenges in integrating nutrition into food policy. This study suggests that this challenge, in part, reflects the historical legacies of strong global consensus regarding a liberal approach to agricultural policy since the 1980s, and of the prior experience of a controlled/ interventionist approach to agriculture in the early years post-independence that did not achieve desired objectives. Despite recognition of the limitations of the current economic paradigm, this perceived failure of state intervention seems to limit willingness to undertake strong intervention in food policy.

Our findings are consistent with previous research that suggests that SAPs and liberalization had a lasting impact on Agriculture policy priorities in Ghana, leading to an emphasis on export-led agriculture.^70^ In addition, the results of this study reflect global findings regarding the impact of policy conditionalities associated with international financial institutions, such as the World Bank and the IMF, on public discourse and beliefs. In Africa and Latin America in the 1980s and Europe and Asia in the 1990s, conditionalities that promoted liberalization have been found to shift policies, as well as discourse, towards a more liberal economic approach over the long term.^21^ In India, economic paradigms have shaped food policy discourse with limited relevance to nutrition,^22^ similar to the historical dissociation of food from nutrition in Ghana and the emphasis on technical solutions to malnutrition seen in this study. This dissociation contributes to ambiguous policy about healthy diets with limited connection to food systems.

The findings of this study also speak to the lasting impact of a narrowly economic paradigm for food security, which focusses only on aggregate food availability with little concern for inequalities and dietary quality.^71^ Food policy paradigms translate into metrics for what success looks like. Across LMICs these metrics have tended to focus on production and reducing hunger.^72^ Such a ‘productivist paradigm’ narrows the scope and imagination for considerations of nutrition, among other social and equity issues, within agricultural policy.^73^ In this study, interviewees talked about ‘success’ in agriculture in relation to the amount produced (including in relation to ‘self-sufficiency,’ but without reference to for whom it was produced), diversification (but without reference to what was produced), price of foods, and farmer access to export markets. In other domains of global health, dominant neoliberal economic paradigms have also resulted in tight, economically-grounded metrics with major implications for the ways in which successful interventions are understood and defined, raising concerns about the potential for innovation and creative risk-taking in addressing major global challenges to be stifled.^74,75^

This study highlights the potential for considering how integration of nutrition into food system policy can also enhance (or at least support) the priorities associated with food policies across sectors. The Agriculture sector in Ghana accounts for one-fifth of GDP, “employs nearly half of the workforce and is the main source of livelihood for the majority of the country’s poorest households.”^76^ Our analysis suggests that lasting policy change to support consideration of nutrition in food policy will only be possible if it (1) identifies specific and feasible points for change, to combat the strong path-dependency of current paradigms, and (2) demonstrates how they contribute to achieving economic, social and environmental sustainability.^77^ As identified in agriculture policy research more broadly, existing beneficiaries of current agricultural policy will be resistant to change.^21^ In addition, in the context of increasingly consolidated supply chains, the power of downstream actors, such as food retailers, in the food system is also growing.^78^ These actors have strong interests in maintaining a status quo in which food is considered primarily as an economic commodity and policy focus remains on enabling business activity.^79^Thus, nutrition-oriented efforts towards food system transformation must be strategic and specific.^80^ For example, existing diet quality indicators (like the Minimum Dietary Diversity for Women) can be applied more rigorously through governmental and independent assessments of food systems,^81^ as can metrics to capture the overall benefits and costs of consuming locally sourced fruits and vegetables compared to importation (such as through development bank ‘score cards’ for nutrition-sensitivity of food systems).^82^ In addition, food value chains analyses can be used to address the underlying determinants of nutrient-poor diets (such as by applying tools like the Global Alliance for Improved Nutrition and Institute of Development Studies’ ‘Nutritious Agriculture by Design’).^83^

Expanding food policy-makers’ consideration of nutrition to ‘nourishing’ food (not simply ‘enough’ food) will require critical engagement on the part of the health sector with the political economy of food systems. Such an expansion will require a new way of thinking about food policy, identified in this study as paradigm-level change (‘third order’ change, to use Hall’s term). Two potential contributors to paradigm-level change relevant to our findings are policy failure and actor influence within institutional structures.^31^ First, policy failure is indicated by the significant economic *as well as* nutritional challenges that were apparent within food system policy, and as such it could be argued that the current paradigm is not delivering. Second, although powerful actors within the agri-food system have a strong interest in maintaining the status quo, there is potential to disrupt policy legacies through creation of new institutional structures and coalitions.^31,84^ In Ghana, this study suggests there is an opportunity to leverage the evident interest in food policy change across sectors and the whole-of-government commitment to addressing all forms of malnutrition to reorient existing institutional structures for nutrition coordination. Below we explore practical implications of these potential avenues for disruption.

###  The Way Forward: Lessons From Historical Policy Legacies

This analysis has highlighted four lessons that could inform advocacy for a more integrated approach to food policy. First, our research suggested that there is recognition among policy-makers across sectors in Ghana that a liberalized economic approach to food policy is limited in its ability to concurrently achieve the objectives of Economic, Agricultural and Health sectors. A wide range of interviewees raised concerns with the current approach to food policy, suggesting that there may be a policy window for reform. This could provide an opportunity for Health sector input on strengthening consideration of nutrition in order to achieve whole-of-government commitment to nutrition. Strategic capacity building for the Health and Nutrition sector would support the Health sector to take up this opportunity.^85^ For example, complementing technical trainings in nutrition with training in political economy and strategies for effective cross sectoral engagement.

 The second lesson is that efforts towards a more integrated approach must take into account the training of food policy-makers (ie, from the Agriculture, Trade and Industry sectors). The investments made in agricultural training as part of the World Bank SAPs in the 1980s have had a major influence on the maintenance of the liberal economic paradigm in the food-related sectors. As such, there needs to be strategic investment in the future policy workforce that has the necessary capacities to develop and maintain an effective integrated approach to food policy. This includes efforts towards curriculum development and integration of cross-disciplinary units of study.

Third, the Health sector needs to be more strategic in fostering a food systems-based approach to addressing the double burden of malnutrition. The consideration – and creation of – incentives for other sectors has long been recognized as critical for supporting meaningful multisectoral action for nutrition.^86^ There was no recognition in the Health sector policies of the current food policy priorities – primarily economic – of other sectors. By explicitly documenting the economic impacts of poor diets, the Health sector could create a stronger rationale for action by the economic sectors that are responsible for food policy. For example, livelihoods, productivity, and the agricultural workforce are all negatively impacted by diet-related NCDs. Thus, there may be an opportunity for the Health sector to support training and skills-building for the agricultural workforce with respect to nutritious food.

Finally, the current policy approach of decentralization appears to be an unrealized opportunity for integrating food and nutrition policy at the local level. The burden of diet-related NCDs and childhood malnutrition on health systems, educational attainment and the workforce – as well as on households and communities – are often most evident at the level of local governments. In addition, the fact that the National Development Plan articulates comprehensive nutrition considerations technically provides ‘top-down’ support for action at all levels of government. Coordinating bodies are critical for ensuring that multisectoral nutrition policy is adopted and implemented effectively.^87^ As such, a mechanism for coordinating the development and implementation of strategies with co-benefits for livelihoods, food security and nutrition (as well as other objectives, such as environmental sustainability) could build on existing local cross-sectoral governance structures, with an understanding that the policy framework would also need to address inequity in terms of fund allocation across the sectors.

###  Study Strengths and Limitations

A major strength of this study is that it situates current calls for ‘food system transformation’ in an historical policy context in which there has been significant attention and deliberate intervention (or not) in the food and agriculture sector– primarily with no consideration of nutrition. Common implications of a historical focus on liberal economic approaches with significant impacts on agriculture have been widely documented across LMIC.^16^ Our intent is that this contextualization of food policy enables public health nutrition actors to engage more strategically with key players in food governance. An historical perspective, in this case examining the post-Independence period in Ghana, can provide points of engagement that focus on revisioning food policy to address multiple objectives: nutrition, livelihoods and environment. It could provide a starting point for food policy-makers to step away from entrenched paradigms to identify where activities and institutional arrangements no longer support national policy objectives.

Methodologically, this study has combined theories of policy learning and policy change with institutionalist theory regarding path dependency to examine the historical antecedents of the current food policy space in Ghana. This combination of theories proved helpful in orienting the analysis of the current policy space in such a way that it was possible to identify key elements of the persistent exclusion of nutrition, and then examine their historical antecedents. In particular, Hall’s conception of policy paradigms as underpinning policy content (instruments and tools) supported the articulation of historical legacies related to policy content and paradigms in an integrated way.^31^

 The study also has several limitations. We examined major events and shifts in policy paradigms and approaches over several decades and thus, the analysis is necessarily high level and focused on understanding the evolution of food policy in Ghana over time with respect to paradigms and objectives. As a result, the study does not address the nuances of food policy development in Ghana or provide a detailed assessment of the impact of food policy on the food system. In addition, although we draw on multiple sources of data, the scope of the project spans economic history, policy analysis and agricultural economics. As such, although we have endeavored to identify all relevant sources of data, there are some that are not included because of difficulties in identifying and accessing them. For example, the FAO Archives were a possible source of data, but we could search only in a limited way due to difficulties encountered with the online platform. We also deliberately excluded monetary policy from the scope of our analysis, even though it has a huge impact on agriculture sector indicators. This was because our focus was not on the status of the agriculture sector, but rather on the prevailing policy priorities and paradigms within and preceding the current policy context.

## Conclusion

 This study has examined historical policy legacies evident in current food policy in Ghana, that help to understand the persistent lack of integration of nutrition considerations despite considerable political commitment to nutrition. These legacies include a strong liberal economic paradigm arising from privatization and liberalization from the early 1980s, an historical experience of ‘failure’ in food policy intervention and an historically narrow focus on food security at an aggregate level. This study suggests that an historical perspective can provide a starting point for food policy-makers to step away from entrenched paradigms, and identify points of engagement for the Health sector to foster revisioning food policy to address multiple national policy objectives, including nutrition.

## Acknowledgements

 We gratefully acknowledge the time and expertise of the interviewees.

## Ethical issues

 This project received ethics approval from the Kwame Nkrumah University of Science and Technology (KNUST) Committee on Human Research Publication and Ethics (reference number CHRPE/RC/029/18).

## Competing interests

 Authors declare that they have no competing interests.

## Authors’ contributions

 AMT and RA conceptualized the study. AMT, ReA, CA, LEA, JW, DS, RoA designed the study and developed the interview tools. AMT, CA, LEA, GM, JW collected the documentary and interview data. All authors contributed to the analysis and preparation of the manuscript.

## Authors’ affiliations

^1^Menzies Centre for Health Policy, School of Public Health, University of Sydney, Sydney, NSW, Australia. ^2^Department of Biochemistry and Biotechnology, Kwame Nkrumah University of Science and Technology, Kumasi, Ghana. ^3^Global Health Governance Group, Usher Institute of Population Health Sciences, University of Edinburgh, Edinburgh, UK. ^4^Médecins Sans Frontières, London, UK. ^5^Centre for Universal Health, Chatham House, London, UK. ^6^Development Policy Centre, Australian National University, Canberra, ACT, Australia. ^7^Kwame Nkrumah University of Science and Technology, Kumasi, Ghana.
